# Dimensional ensemble and (topological) fracton thermodynamics: the slow route to equilibrium

**DOI:** 10.1038/s41598-019-49141-w

**Published:** 2019-09-05

**Authors:** J. C. Flores

**Affiliations:** Departamento de Física, FACI, Universidad de Tarapacá, Casilla 7-D, Arica, Chile

**Keywords:** Physics, Thermodynamics

## Abstract

The use of the dimensional-ensemble becomes compulsory when spatial dimensions are not well defined. Consequently, apart from temperature, thermodynamic equilibrium requires an additional configurational parameter. Two representative cases are considered in detail: oscillators with undefined spatial dimension and topological fractons. Spatial dimension and energy are determined as a function of temperature in both cases. At low temperatures, specific heat behaves exponentially, meaning it creates a slow route to equilibrium. In accordance with experiments, calculations suggest that the spatial dimension diminishes when temperature decreases. Parameter values are computed using data obtained from almost two-dimensional graphene and porous compounds.

## Introduction

Decades of study uncovered the thermal properties of glasses, particularly their exponentially slow time behaviour in reaching equilibrium at low-temperatures^1^. This behaviour can be natural in weakly disordered systems where, in the core of the Lifshitz tails^2–5^, the Helmholtz free energy behaves like $\Delta F\propto exp(\,-\,\sigma /kT)$ as well as for some gases at low temperatures^6^ (*σ* amount of disorder and *T* temperature). However, as pointed out by Chamon^7^, thermodynamic glass behaviour may exist in ordered systems. That is, near *T* ~ 0^+^ some ordered systems reach equilibrium slowly. Along the same lines, another exactly solvable example was developed by Prem *et al*.^8^ where, always at low temperature, the thermodynamics of fractal excitations^9,10^ possess specific heat *C* that decreases exponentially with temperature as

1 $$C\propto {(\frac{DW}{kT})}^{2}{\exp }(-\frac{DW}{kT}),$$

where *W* is an energy parameter related to excitation creation with the spatial dimension being *D* = *0*.*5*. Indeed, using Eq. (1), as long as the system receives externally a fixed rate of heat flux *ϕ*, the solution of the balance energy equation, $C\frac{dT}{dt}=\varphi$ (*t* time), possesses a slow logarithmic behaviour for *T*(*t*). Additionally, always in the sense of ordered systems, Einstein’s model for the heat capacity of solids^6,11–13^ at low temperatures has an exponential behaviour similar to (1) involving specific applications^14–17^ (revisited in Section III).

Fractals are characterized by scale invariance and non-integer dimensions^10,18,19^. Much effort has been spent applying fractal concepts to glasses, amorphous and, in general, disordered systems. Particularly interesting is the work of Tyurin *et al*.^20^ on compound semi-conductors and correlating dimension and temperature^21^. Here, these concepts are used within the dimensional ensemble framework. Nevertheless, other explanations can eventually be formulated.

In this work, discussions turn around fractons, i.e., physical excitations on systems with non-integer spatial dimensions. Actually, here the dimension value comes from the thermodynamics average in the dimensional ensemble and is related to finite temperature. The dimensional ensemble comes from the realization that not only the energy but also the spatial dimension is hardly ever measured directly. It should be noted that an ensemble corresponds formally to virtual repetitions, at different states, of a given physical system. In the present case, these states are indexed by the energy and the spatial dimension.

The behaviour related to Eq. (1) must be framed in terms of a statistical ensemble where spatial dimension *D* is, apart from energy, an undefined quantity (Section II). As an example, consider a particle of mass *M* transiting between two subsystems (boxes) of dimension *D* = 1 and *D* = 2, where the energy of the particle^22^ is $\frac{{\hslash }^{2}}{2M{L}^{2}}{n}^{2}$ and $\frac{{\hslash }^{2}}{2M{L}^{2}}({l}^{2}+{m}^{2})$, respectively, with *L* a length defining the size and *n*, *l*, *m* integers. When, from the first subsystem (e.g., *n* = 5), the particle is transferred to the other (e.g., *l* = 3; *m* = 4 or *l* = 4; *m* = 3), it possesses the same energy but attains greater degeneracy or entropy *S*. Then, when the particle goes to the subsystem of a major dimension, the Helmholtz free energy $\Delta F=-\,T\Delta S$ decreases. In equilibrium, and for fixed internal energy, this diminution must be compensated with a term related to the change in dimension Δ*D*. That is, consider the conjecture

2 $$W\Delta D-T\Delta S=0,$$

where *W* is the additional parameter necessary to define equilibrium, used explicitly in ref.^8^, it is the configuration parameter. In the dimensional ensemble framework, applications of Eq. (2) are developed below for oscillators (Section III) and fractons (Section IV). More important, conjecture Eq. (2) becomes deeply related to the definition of this ensemble in the next section.

At this point, the question of experimental evidence supporting the idea of a dimensional-ensemble arises. In fact, excitations in fractal systems are not entirely developed in the literature from a thermodynamics point of view, although it is an important subject with many applications involving physics, engineering, and possibly chemical processing. Further, to the best of my knowledge, this is the first work concerning explicitly thermodynamic aspects of systems with uncontrolled spatial dimension.

## Dimensional Ensemble

In general terms, consider a system composed of non-interacting particles (eventually, subsystems). For every particle, the energy-level is *E*_*i*_ at dimension *D*_*i*_. Moreover, let *p*_*i*_ be the probability of a particle having energy *E*_*i*_ (average 〈*E*〉) at dimension *D*_*i*_ (average 〈*D*〉). With these constraints, the entropy function technically defined by Jaynes^23^ $(\sum \{{p}_{i}\,{ln}({p}_{i})-\beta {p}_{i}{E}_{i}-\gamma {p}_{i}{D}_{i}\})$ can be extremalized, and this standard procedure gives the partition function

3 $$Z=\sum _{\{i\}}exp(-\beta {E}_{i}-\gamma {D}_{i}),$$

where the parameters *β* and *γ* correspond to Lagrange multipliers that determine the equilibrium state, and the summation runs over all microstates. Note that *Z* can be rewritten as

4 $$Z=\sum _{{D}_{i}}{Z}_{i}exp(-\gamma {D}_{i}),$$

where *Z*_*i*_ is the partition function in the canonical ensemble, which depends on particular dimension *D*_*i*_. To clarify briefly the above procedure connected to the deduction of Eqs (3) or (4) for the partition function, consider that formally this procedure is similar to the case of the grand canonical ensemble with two thermodynamic parameters *T* and chemical potential *μ*. A similar situation exists for the isobaric ensemble with *T* and pressure *P* as thermodynamic parameters^24^. That is, there are ensembles where not only the energy is undefined and which require more than one parameter aside from temperature. Naturally, in the thermodynamic limit, these ensembles are expected to be equivalent^6,11,12^ almost for any system.

The average energy and average dimension can be formally obtained from Eq. (4) as

5 $$\langle E\rangle =-\,\frac{\partial }{\partial \beta }\,{ln}(Z),$$

6 $$\langle D\rangle =-\,\frac{\partial }{\partial \gamma }\,{ln}(Z).$$

Additionally, as expected, fluctuations are related to second derivatives of *ln*(*Z*). For practical reasons, define the Lagrange multiplier *W* (see Eq. (2)), through

7 $$\gamma =W\beta .$$

Now I consider briefly the interrelation between thermodynamic variables like free energy, entropy and so on. In fact, replacing directly the equilibrium expression for probability ${p}_{i}=\frac{1}{Z}exp(\,-\,\beta {E}_{i}-\gamma {D}_{i})$ onto entropy $S=-\,k\sum {p}_{i}\,\mathrm{ln}({p}_{i})$ then free energy $F=-\,\frac{1}{\beta }ln(z),$ internal energy 〈*E*〉, and dimension 〈*D*〉 are related through the expression (per particle):

8 $$F=\langle E\rangle +W\langle D\rangle -TS,$$

where the averaged dimension 〈*D*〉 does not necessarily take integer values. Note that the classical relationship^6,11,12^ between thermodynamics variables is formally re-obtained when *W* = 0.

Additionally, the vibrational modes with a density of states $\propto \,{E}^{D-1}$ have an energy expectation 〈*E*〉 = *kT*〈*D*〉. Moreover, as occurs with the definition of temperature $kT=\frac{\partial S}{\partial E}$ at volume constant, Eq. (8) suggests that in the microcanonical ensemble $k\gamma =\frac{\partial S}{\partial D}$.

Two final points are important: (a) In this work, spatial dimensions take discrete finite values {0, *D*_1_, *D*_2_, …}, where the dimension *D* = 0 contains explicitly trapped excitations and, accordingly, is related to bad thermodynamic conduction. (b) I insist, the derivation of (8) comes from the entropy functional with constraints on the energy and the spatial dimension; nevertheless, Eq. (2) is non-standard. In fact, it is a conjecture defining the dimensional ensemble.

## Oscillators in the Dimensional Ensemble

The partition function of a one-dimensional oscillator^11,12^ with intrinsic frequency *ω*_*o*_ is proportional to ${(1-{e}^{-\hslash {\omega }_{o}\beta })}^{-1}$ and easily generalizable to arbitrary dimensions $({(1-{e}^{-\hslash {\omega }_{o}\beta })}^{-D})$. Corrections due to the zero-point energy will be briefly touched at the end of this section.

Assume two dimensions 0 and *D*; then from Eq. (4), the partition function is

9 $$Z=1+\frac{{e}^{-\gamma D}}{{(1-{e}^{-\hslash {\omega }_{o}\beta })}^{D}}.$$

From Eq. (6), the average dimension becomes

10 $$\langle D\rangle =D\frac{1}{1+{e}^{\beta WD}{(1-{e}^{-\hslash {\omega }_{o}\beta })}^{D}}$$

when $T\to \infty$, $\langle D\rangle \to D$. In contrast, when $T\to 0$, $\langle D\rangle \to 0$ $(W\ne 0)$ is valid for high-frequencies $\hslash {\omega }_{o} > kT$ and useful for thermodynamic perturbations. Importantly and as previously mentioned, dimension zero is correlated with deeply trapped excitations.

From Eq. (5), the internal energy turns out in this case to be

11 $$\langle E\rangle =\hslash {\omega }_{o}D\frac{1}{{e}^{\hslash {\omega }_{o}\beta }-1},$$

where relation (7) was explicitly used in both calculations of Eqs (10) and (11). Note that (11) is valid for any dimension values (not only for the bi-evaluated case 0 and D).

Up to this point, the discussion is similar to the two-energy level case discussed in statistical physics textbooks. So, why is the present approach relevant? Moreover, note that ensembles are equivalent in the thermodynamics limits. Nevertheless, for a given thermodynamics system, calculations are more easily realized inside a given ensemble than another. For instance, the Fermi–Dirac statistic is easily obtained in the grand-canonic rather than in the micro-canonic. To go deeper, and as mentioned above, consider the relation between energy (work) and pressure ($E=PV$) obtainable for instance in the canonic after a series of steps. Think through the isobaric (P = constant) ensemble, where probability $p \sim exp(\,-\,\beta E-P^{\prime} V)$ and the volume is undefined, as is the energy. Assume a two-level system with volume *V*_1_ and *V*_1_ where $Z=exp(\,-\,\beta E-P^{\prime} {V}_{2})+exp(\,-\,\beta E-P^{\prime} {V}_{2})$. As long as in this ensemble $\langle V\rangle =-\frac{\partial }{\partial P^{\prime} }lnZ$ and $\langle E\rangle =-\frac{\partial }{\partial \beta }lnZ,$ then 〈*E*〉 = P〈*V*〉 is quickly obtained (pressure $P={\beta }^{-1|}P^{\prime}$). This formal expression (E = PV) can also be computed from the canonic where the volume is fixed.

Regarding the oscillator, at low temperatures the energy behaves like (*W* ≠ 0)

12 $${\langle E\rangle }_{T\to 0}\approx \hslash {\omega }_{o}D{e}^{-(\hslash {\omega }_{o}+WD)/kT},$$

and necessarily, the specific heat exhibits exponential decay with temperature. The formal case *W* → 0 contains the usual exponential behaviour mentioned in the Introduction and related to Einstein’s description of solids, specifically, a glass behaviour as in Eq. (1).

In the low-temperature regime, we have from Eq. (10) $\gamma  \sim \frac{1}{D}ln\frac{D}{\langle D\rangle }$. Then, following Valalaki and Nassiopoulou^19^ with 〈*D*〉 ~ 1.822 and *D* = 2 for vibrations in porous Si compounds around 10°*K*, an estimation gives *γ* ~ 4.6606 × 10^−2^ or $W \sim 6.43\times {10}^{-24}$ [J] corresponding approximately to 4 × 10^−5^ [eV] in this instance. Naturally, other justifications can explain the mentioned non-integer dimension. It must also be mentioned that in porous Si compounds, thermal conductivity diminishes from^19^ 0.2 [W/mK] (at 300°K) to 0.04 [W/mK] (at 10°K). This can be partly explained with the diminishing of the averaged dimension at low temperature, i. e., it approaches zero as mentioned previously (see Eq. 10).

From Eq. (8), it is expected that the entropy has two contributions: (a) vibrational 〈*E*〉/*T* and (b) dimensional (or configurational) *W*〈*D*〉/*T*. Then, from Eq. (11), the ratio between these two contributions becomes $\sim \frac{1}{{e}^{\hslash {\omega }_{o}\beta }-1}$. At low temperatures, this ratio behaves exponentially small and, necessarily, configurational entropy exceeds vibrational entropy, as occurs for metallic glasses^25^. At the opposite limit of high temperature, vibrational entropy is larger than configurational entropy.

Three final remarks:

- The oscillator of frequency *ω*_*o*_ can be viewed as a wave mode (i.e., $\omega =c|\overrightarrow{K}|$); then, for a fixed temperature, expression (10) represents the average fractional dimension for a wave of wavelength *λ* in the dimensional ensemble.
- Incorporating zero-point energy in the average dimension, Eq. (10) is achieved by the formal correspondence: $W\to (W+\frac{\hslash {\omega }_{o}}{2})$.
- Additionally, incorporating zero-point energy in the internal energy, Eq. (11), requires $\langle E\rangle \to (\langle E\rangle -\frac{\hslash {\omega }_{o}}{2}\langle D\rangle )$.

When temperature approaches zero, dimension 〈*D*〉 diminishes exponentially (*W* ≠ 0, Eq. (10)). In consequence, zero-point energy $(\hslash {\omega }_{o}/2)\langle D\rangle$ reduces in line with temperature. On the other hand, Casmir’s forces are deeply related to the zero-point energy^26–28^, including realizations in mesoscopic systems^29–31^. Consequently, at sufficiently low temperatures, a summation like $\sum _{K}\frac{\hslash \omega (K)}{2}\langle D\rangle$ can be eventually non-divergent.

The next section considers topological excitations, where energy-level variations are not relevant.

## Specific Heat for Topological Fractons

Consider a system composed of two subsystems of intrinsic dimensions *D*_1_ and ${D}_{2}={D}_{1}+\Delta D$ and accessible only to a relevant energy *E*_*o*_. Specifically, there are no transitions between energy levels but only between dimensional spaces as in the example mentioned in the Introduction.

From Eq. (4), the partition function, in this instance, is written as

13 $$Z={e}^{-\beta {E}_{o}}({e}^{-W\beta {D}_{1}}+{e}^{-W\beta {D}_{2}}).$$

From Eqs (6) and (7), the average spatial dimension is found to be

14 $$\langle D\rangle ={D}_{1}+\frac{\Delta D}{{e}^{W\beta \Delta D}+1}$$

with range *D*_1_ ≤ 〈*D*〉 ≤ *D*_2_ for different values of *T*. The system supports, theoretically, negative temperatures because there is a formal energy maximum^6,12^ of *WD*_2_. Additionally, note that this average dimension 〈*D*〉 (Eq. 14) does not contain intrinsic scale parameters. Indeed, *γ* = *Wβ* is a parameter defining the equilibrium and is not intrinsic as occurs, for instance, with *ω*_*o*_ in Eq. (10).

**Figure 1:**
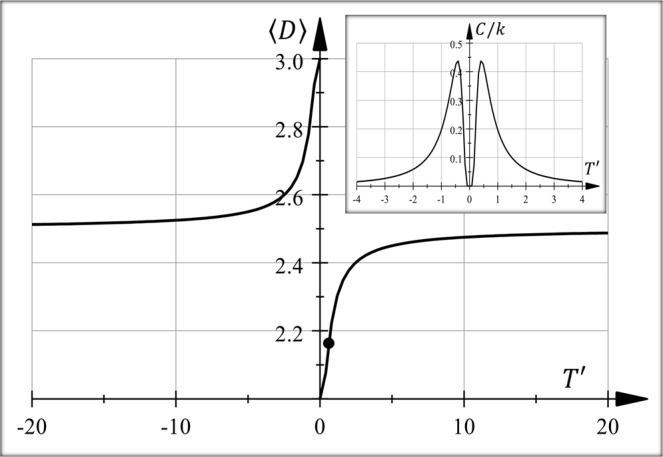
Average dimension 〈*D*〉 as a function of normalized temperature $T^{\prime} =kT/W\Delta D$ for topological fractons with *D*_1_ = 2 and *D*_2_ = 3 (eventually, the temperature can be negative). Formally, the black dot marks where ripples on graphene occur at 300°*K*. The parameters defining the curve, *T* and *W*, are only thermodynamic because there is no intrinsic parameter defining scales. Inset: specific heat as a function of normalized temperature *T*′. Near-zero temperature, the specific heat decays exponentially in accordance with Eq. (16). This way, at low-temperatures, topological fractons approach equilibrium slowly.

Figure 1 shows the average dimension 〈*D*〉 as a function of the dimensionless temperature $T^{\prime} =kT/W\Delta D$ when *D*_1_ whe and *D*_2_ and. For high positive temperatures, the spatial dimension goes to 2.5. Theoretically graphene is a sheet of carbon atoms with two-dimension Dirac-like excitations. Ripples are associated with soft structures for the most part with instabilities at dimension two where they range between^32^ 2 ≤ D ≤ 3. Further, these structure distortions can be related to thermal processes^32,33^. At 300°*K*, a dimension^21,26^ of 〈*D*〉 ~ 2.1827 gives a value *W* ≈4.49× 10^−21^ [J] or 2.81 × 10^−2^ [eV].

From Eq. (2), specific heat $C=W\frac{\partial \langle D\rangle }{\partial T}$ for these topological fractons is

15 $$C=k{(\frac{W\Delta D}{2kT})}^{2}\frac{1}{{(cosh(W\Delta D/2kT))}^{2}},$$

and, at low-temperatures *kT* ≪ *W*Δ*D* becomes

16 $${C}_{T\to {0}^{+}} \sim k{(\frac{W\Delta D}{2kT})}^{2}{e}^{-W\Delta D/kT},$$

in accordance with the expression in ref.^8^ where Δ*D* = 0.5.

Figure 1 (inset) shows a plot of the heat capacity (Eq. (15)) for these excitations as a function of the dimensionless temperature $T^{\prime} =kT/W\Delta D$. Around *|T|* ~ 0, the behaviour is an exponential decay following Eq. (1). Consequently, as for glasses, equilibrium is slowly reached for topological fractons (see Introduction).

Finally, dimension fluctuations can be evaluated as long as ${\langle D\rangle }^{2}-{\langle D\rangle }^{2}=\frac{{\partial }^{2}}{\partial {\gamma }^{2}}ln(z)$. Then

17 $${\langle D\rangle }^{2}-{\langle D\rangle }^{2}=\frac{{(\Delta D)}^{2}}{4\,{\cosh }(\frac{\gamma \Delta D}{2})},$$

going to zero when Δ*D* → 0. As a function of temperature (*γ* = *W*/*kT*), fluctuations go to zero when T → 0 (*W* ≠ 0).

## Conclusions and Outlook

Thermodynamic systems, for which the spatial dimension is not well defined, can be described through the dimensional ensemble, which was inferred from appropriate constraints on the entropy functional^23^. Equilibrium becomes characterized by temperature *T* and configurational parameter *W* (or *γ*), related to dimension variations. Thermodynamic fractons are excitations in these systems with an averaged dimension depending on temperature.

For example, an oscillator was considered having an unspecified dimension of either 0 or *D*. In the limit of low-temperature *T*, the average dimension goes to zero; for large *T*, the dimension goes to its maximum value *D*. Estimations of thermodynamic parameters for compound-semiconductors were realized. Moreover, from a generic point of view, the behaviour of metallic glasses was also considered.

The focal subject in this work was topological fractons. These are excitations for which quantum energy levels are frozen, but they can change dimension (*D*_1_ or *D*_2_). The average spatial-dimension of these excitations is characterized only by the thermodynamic equilibrium parameters *kT* and *W*. The system admits positive and negative temperatures. The specific heat for these thermodynamic excitations has a decreasing exponential dependence at low temperature. Consequently, topological fractons slowly reach thermodynamic equilibrium, i.e., a glass-like behaviour. Evaluations for ripples in graphene were briefly realized.
