# Publisher Correction: Dimensional ensemble and (topological) fracton thermodynamics: the slow route to equilibrium

**DOI:** 10.1038/s41598-020-68762-0

**Published:** 2020-07-22

**Authors:** J. C. Flores

**Affiliations:** Departamento de Física, FACI, Universidad de Tarapacá, Casilla 7-D, Arica, Chile

Correction to: *Scientific Reports* 10.1038/s41598-019-49141-w, published online 05 September 2019

The original version of this Article contained missing angle brackets in equation 11. This has now been corrected in the PDF and HTML versions of the Article.

